# Comparative study on the thermal runaway characteristics of Li(Ni_x_Co_y_Mn_z_)O_2_ batteries

**DOI:** 10.1016/j.heliyon.2024.e31203

**Published:** 2024-05-14

**Authors:** Ningning Wei, Fengqin Zhang, Wei Zhang, Xin Li

**Affiliations:** a School of Urban Rail Transit, Shandong Polytechnic, Jinan 250000, China; b Department of Rail Transit, Hebei Jiaotong Vocational and Technical College, Shijiazhuang 050035, China

**Keywords:** Lithium-ion battery, Thermal runaway, Gas production, Gas release characteristics, Thermal characteristics, Flammability limit

## Abstract

Lithium-ion batteries (LIBs) generate substantial gas during the thermal runaway (TR) process, presenting serious risks to electrochemical energy storage systems in case of ignition or explosions. Previous studies were mainly focused on investigating the TR characteristics of Li(Ni_x_Co_y_Mn_z_)O_2_ batteries with different cathode materials, but they were conducted in isolation. In this study, the thermal runaway characteristics of prismatic cells that use Li(Ni_x_Co_y_Mn_z_)O_2_ (with x ranging from 0.33 to 0.9) cathode materials in an inert environment, which are commonly used or proposed for energy storage applications, are examined. The findings of this research show that the normalized gas generation rate remains consistent, regardless of the battery capacity or experimental chamber volume, with a value of 0.1 ± 0.03 mol∙Ah^⁻1^. High-capacity cells have short jetting durations, and a high nickel content leads to increased mass loss rates. The flammability limits of the gases expelled during thermal runaway, represented by the lower flammability limit (LFL), remain stable at 8 ± 1.8 % with minimal variations. However, the upper flammability limit (UFL) varies significantly, ranging from 30 % to 60 %. Increasing the battery capacity or reducing the experimental chamber volume increases the explosion index. The explosive, combustibility, and jetting duration characteristics of the emitted gases from five different battery chemical compositions provide valuable insights for risk assessment in future electrochemical energy storage systems.

## 1 Introduction

The ternary battery, specifically the Li(Ni_x_Co_y_Mn_1-x-y_)O_2_ (NCM) ternary battery, is increasingly regarded as a pivotal technology within the realm of electrochemical energy storage. This category of batteries amalgamates the advantages of metals such as nickel, manganese, and cobalt, exhibiting high energy density, prolonged cycle life, and commendable safety performance [1]. The advantages of lithium-ion batteries are particularly evident in the context of renewable energy utilization and the electrification of transportation [[2], [3], [4]]. Presently, across numerous countries and regions worldwide, the NCM ternary battery is increasingly acknowledged as the preferred electrochemical energy storage systems (ESSs) [5].

The selection of batteries for energy storage facilities is a complex decision-making process, where the importance of different parameters depends on specific application scenarios. One crucial factor in all energy storage contexts is the thermal stability of batteries [6]. This factor includes the challenge of avoiding TR under thermal, electrical, and mechanical harm and the management of energy release during the TR process [1,[7], [8], [9], [10]]. Electrolyte vapor, which is a combustible mixture, is emitted by batteries during TR; it is both flammable and prone to explosion [11]. Gas jet explosions, which release high levels of energy, are the most likely cause of casualties [7,12,13]. Given the high energy densities of energy storage systems, the choice of lithium-ion batteries should prioritize safety and electrical performance parameters [[14], [15], [16], [17], [18], [19]].

The safety standards for energy storage systems (ESSs) and equipment, as outlined in UL9540A-2019, emphasize the importance of assessing the generation of TR gases, combustion rates, explosion overpressures, and fire suppression mechanisms to ensure the safety of energy storage batteries [5]. In order to mitigate the risk of fires and explosions in energy storage facilities, it is imperative to enhance real-time monitoring and maintenance of battery systems to promptly detect anomalies. Employing fire-resistant isolation devices, automatic fire suppression systems, and other protective measures can effectively reduce the likelihood of fires and explosions, ensuring the stability and reliability of these systems during prolonged operations [20,21]. The implementation of these comprehensive measures significantly contributes to minimizing the hazards associated with fires and explosions in energy storage facilities, thereby ensuring the safety and sustainability of energy storage systems [22].

Until now, three distinct types of experimental environments have been identified for investigating thermal runaway [23]:

Approach 1: Open environment experiments, such as those conducted by Ribière et al. [24] and Wang et al. [25], involve investigating battery thermal runaway ignition events and conducting thermal diffusion experiments of battery modules. This experimental approach affords a more realistic and practical simulation, capturing thermal runaway scenarios that batteries may encounter during actual usage. By permitting on-site monitoring and observation, researchers can contemporaneously gather data on the runaway process, facilitating an in-depth understanding of the mechanisms and characteristics involved. Moreover, the open environment offers flexibility, allowing for easier adjustment and control of experimental conditions, encompassing factors such as temperature, humidity, and atmosphere, thus better accommodating the specific requirements of the research at hand.

Approach 2, Semi‒open experimental experiments, such as those conducted by Liu et al. [26], Zhou et al. [27] and Larsson et al. [28]. This experimental approach, while simulating the thermal runaway process of batteries, effectively mitigates experimental risks through the implementation of specific protective measures. The semi-open environment allows researchers to conduct experiments under relatively controlled conditions, adjusting parameters such as temperature and humidity for greater flexibility in addressing specific research requirements. In contrast to approach 1, within a semi-closed environment, the combustion atmosphere is real-time air, potentially resulting in the test gas being mixed with other gases present in the air.

Approach 3, Closed inert space experiments, such as those conducted by Vijay Somandepalli et al. [29] and Wei et al. [30]. This experimental setting provides highly controlled conditions, effectively mitigating experimental risks and ensuring the safety of the experimental process. By manipulating the atmospheric composition within the environment, such as employing inert gases like nitrogen or argon to reduce oxygen concentration, the risk of fire or explosion can be significantly diminished. A sealed inert environment further affords stable experimental conditions, allowing for the observation of in-situ venting characteristics during thermal runaway, thereby enhancing the reliability and reproducibility of experimental outcomes.

To date, significant research has been devoted to examining the TR properties of Li(Ni_x_Co_y_Mn_z)_O_2_ batteries with diverse cathode materials. However, it is worth noting that the majority of these investigations are rather disconnected and lack coordination. In contrast, the behaviors of ternary prismatic batteries that possess different cathode materials during TR events are scrutinized. The research setting is arranged in a fixed-volume adiabatic experimental chamber (AEC) with a 1000-liter capacity, where occurrences of battery thermal runaway occur in an inert atmosphere. Comparative analysis is carried out on the thermal runaway (TR) characteristics of batteries using various cathode materials, including Li(Ni_x_Co_y_Mn_z_)O_2_ with x values ranging from 0.33 to 0.9. The study addresses essential parameters, including the flammability limits of gases emitted during thermal runaway (TR), the duration of jetting during TR, and the explosion indices associated with the emitted gases. The primary aim of this research is to elucidate the fundamental mechanisms governing hazard formation. Moreover, the study seeks to provide valuable recommendations for selecting suitable battery chemistry and devising relevant protective measures. The findings of this research contribute to establishing a reliable theoretical foundation for enhancing the protection of energy storage systems (ESSs) and ensuring the safe utilization of batteries within these systems.

## 2 Experimental setup

### 2.1 Battery samples

The experimental design involved five different battery types, each with distinct electrochemical properties. These batteries used different cathode materials, including Li(Ni_1/3_Co_1/3_Mn_1/3)_O_2_ (NCM111), Li(Ni_0.5_Co_0.2_Mn_0.3)_O_2_ (NCM523), Li(Ni_0.6_Co_0.2_Mn_0.2)_O_2_ (NCM622), Li(Ni_0.8_Co_0.1_Mn_0.1)_O_2_ (NCM811) [30], and Li(Ni_0.9_Co_0.05_Mn_0.05_)O_2_ (NCM9 0.5 0.5). The battery information, as provided by the battery manufacturers, is presented in Table 1. The charging and discharging procedures were executed using the Neware CT-4002-5V100A-NA charge/discharge system, characterized by a voltage/current precision of ±0.1 % FS and a power precision of ±0.2 % FS. All test cells underwent constant current discharge at a rate of 1/3C until reaching the corresponding lower cutoff voltage. Subsequently, they underwent constant current-constant voltage charging at a rate of 1/3C until attaining the respective upper cutoff voltage. Each battery specimen underwent three charge-discharge cycles. Prior to conducting the TR test, it was ensured that each battery's state of charge (SOC) was charged to 100 %.

**Table 1:** Battery sample specifications.

Sample cathode	NCM111	NCM523	NCM622	NCM811	NCM9 0.5 0.5
Mass (g)	850	887	862	3420	2450
Voltage (V)	2.8–4.3	2.8–4.3	2.8–4.3	2.8–4.3	2.8–4.3
Geometry (mm)	170 × 20× 110	148 × 27× 102	148 × 27× 96	350 × 35× 120	303 × 34× 100
Shape	Prismatic	Prismatic	Prismatic	Prismatic	Prismatic
Shell Material	Al alloy	Al alloy	Al alloy	Al alloy	Al alloy
Capacity (Ah)	40	50	50	256	186.5
Anode	Graphite	Graphite	Graphite	Graphite	Graphite
Jellyroll	2	2	2	2	2
Cathode current collector	Al foil	Al foil	Al foil	Al foil	Al foil
Anode current collector	Copper foil	Copper foil	Copper foil	Copper foil	Copper foil

### 2.2 Experimental methods

**Fig. 1:**
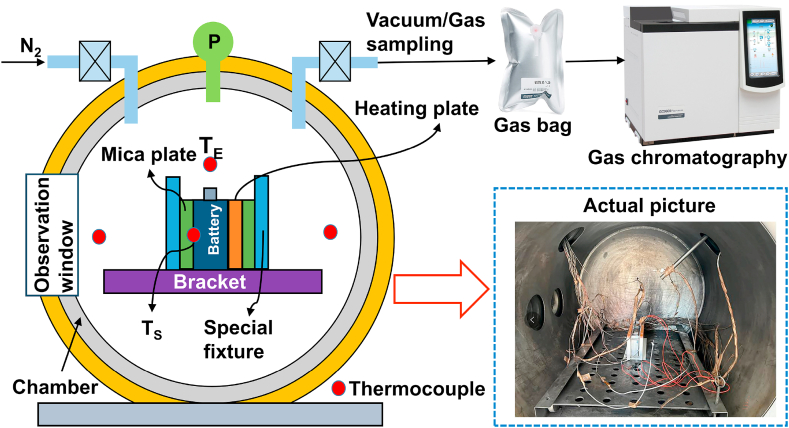
Constant volume pressure vessel for gas characterization.

**Fig. A1:**
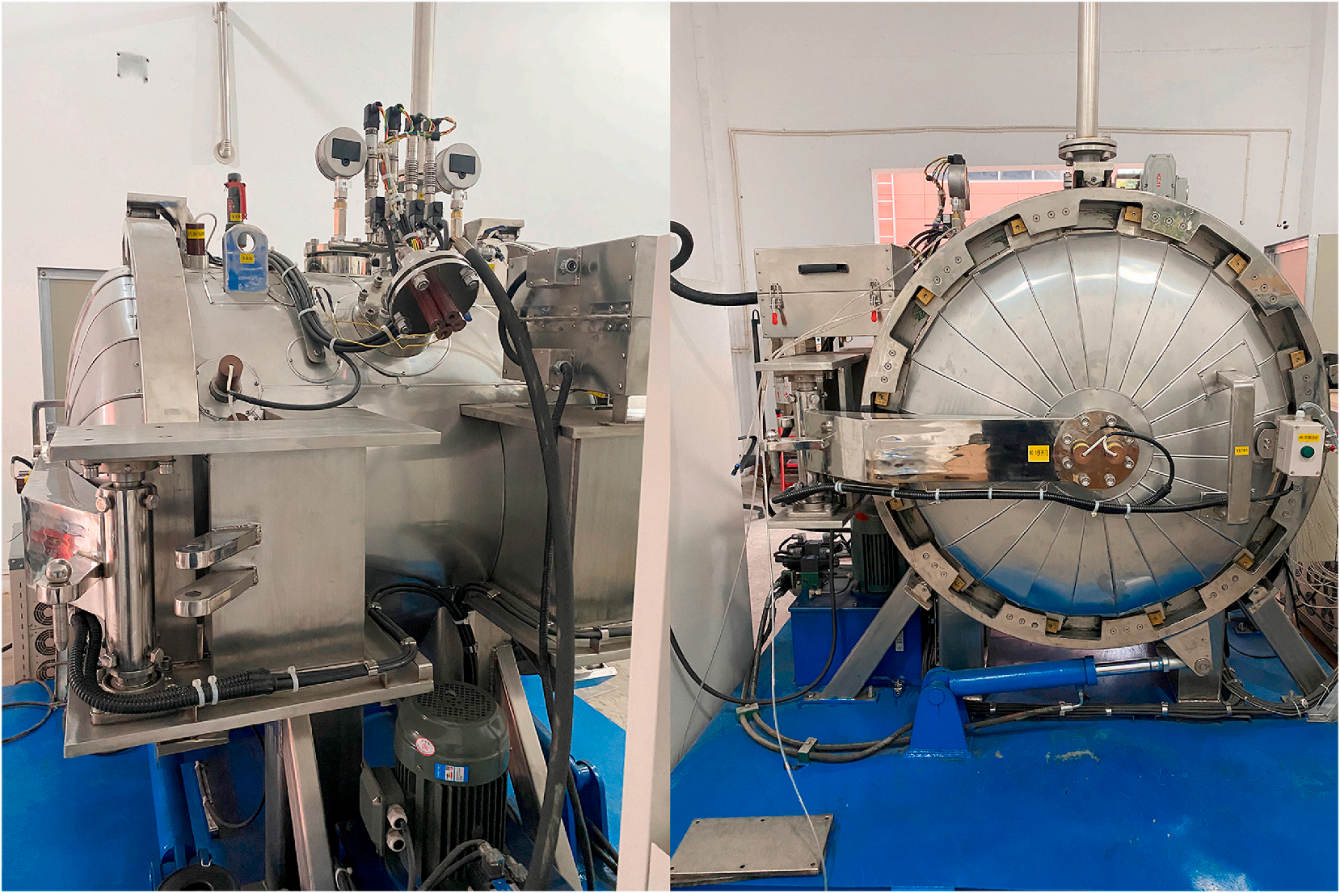
Picture of the experimental cabin.

The battery fixture and the AEC used for thermal runaway (TR) testing of batteries are shown in Fig. 1 and Figure A1 [23,30,31]. This experimental chamber was part of the lithium-ion battery cell/module safety testing apparatus developed by Professor Minggao Ouyang's research group at Tsinghua University. The chamber included a vacuum pump, pressure sensors, temperature sensors, a data acquisition system, and inlet and outlet ports. The internal volume of the chamber was 1000 L, and it could withstand an internal pressure reaching 10 MPa. Multiple K-type thermocouples and four pressure sensors were installed inside, along with two conductors for real-time battery voltage measurements. Thermocouples were employed to monitor temperature variations at various points on the battery surface and changes in ambient temperature during the thermal runaway process. Simultaneously, pressure sensors detected alterations in internal pressure within the experimental chamber throughout the course of the thermal runaway. Both thermocouples and pressure sensors operated at a sampling frequency of 10 Hz.

The batteries were situated at the central position within the experimental chamber, with thermocouples arranged according to the configuration depicted in Fig. 1. The initial temperature of the chamber was ensured to be 25 °C. A 500-W constant power heating plate was attached to one surface of the battery for heating and inducing TR using a lateral heating approach. The gas displacement procedure for the experimental chamber involved evacuating the chamber gas pressure to 15 kPa using a vacuum pump, followed by nitrogen injection until reaching standard atmospheric pressure. This cyclic process was repeated at least three times to ensure that the air content within the experimental chamber remained below 1 %. The heater was first activated to increase the battery temperature to the TR point and then deactivated. The criterion for gas collection was established based on the stability of gas pressure within the experimental chamber (|dp/dt|<0.2 kPa/s, sustained for 5 s or more). Utilizing data acquired from pressure sensors, gas production was computed based on the ideal gas law, as represented by Equation (1) [5]. As depicted in Fig. 1, post-thermal runaway gases were collected using a sampling bag, and qualitative and quantitative analyses of the gases released during the TR process were performed using Gas Chromatography (GC). After the experiment, TR products from the battery, primarily particulate matter, were collected.

(1) $$n_{\text{gas}}=\frac{P_{\text{gas}}\cdot V_{\text{container}}}{R\cdot T_{\text{gas}}}-\frac{P_{0}\cdot V_{\text{container}}}{R\cdot T_{0}}$$

In this experiment, the Gas Chromatography-Mass Spectrometry (GC-MS) instrument utilized was the GCMS-QP2020NX model. The GC-MS-QP2020 NX is a gas chromatography-mass spectrometry (GC-MS) instrument that combines the separation capabilities of gas chromatography with the mass analysis capabilities of a quadrupole mass spectrometer. The instrument's scanning rate can reach 20,000 U/sec, with a sensitivity of Signal-to-Noise ratio (S/N) ≥ 185 [23].

## 3 Results and discussion

### 3.1 Characteristics of gas production during thermal runaway

**Fig. 2:**
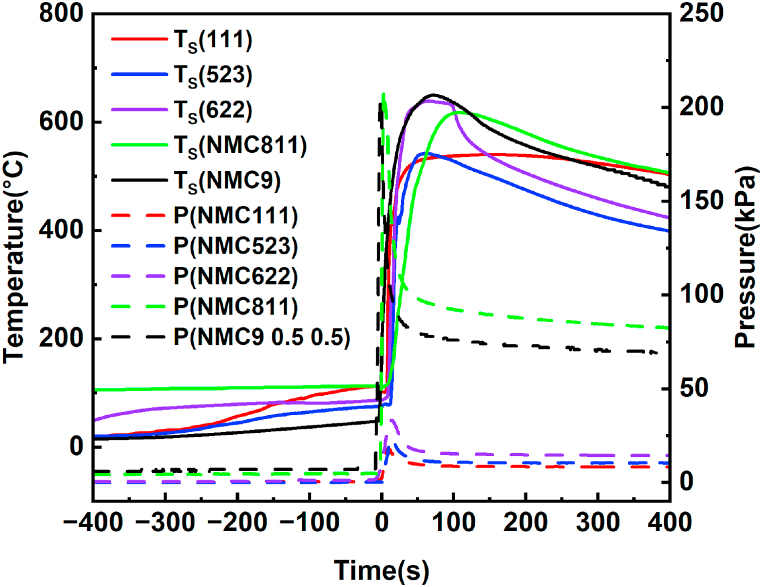
Variations in T_S_ and pressure vs. time.

**Fig. A2:**
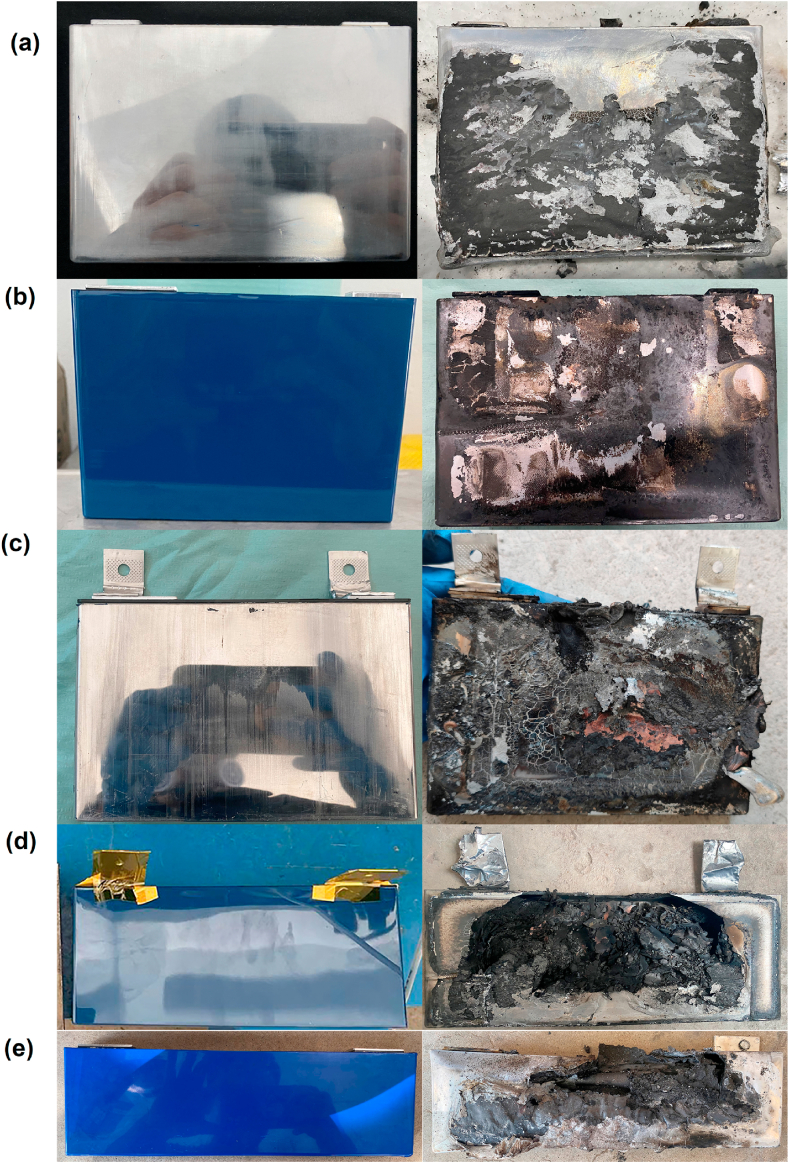
Battery photos: before TR (the left) and after TR (the right), (a)NCM 111, (b)NCM 523, (c)NCM 622, (d)NCM 811, (e)NCM 9 0.5 0.5.

Photographs depicting the battery before and after thermal runaway are presented in Figure A2. In accordance with Section 2.2 and as shown in Fig. 1, various parameters were measured during the TR experiments. These parameters included battery voltage (V), surface temperature (T_S_), experimental chamber temperature (T_A_), and pressure (P). Fig. 2 displays the recorded surface temperatures (T_S_) and pressures (P) collected during the TR test of the battery. The zero-time reference was denoted when the voltage sharply declined to zero. Fig. 2 illustrates that the rise in internal pressure precedes the voltage drop and temperature elevation, acting as an early indicator of TR in a sealed environment. Furthermore, Fig. 2 illustrates the trends in battery surface temperature elevation induced by TR under the same triggering conditions. The arrangement of these trends is as follows: NCM9 0. 5 0.5 > NCM622 > NCM811 > NCM523 > NCM111.

Fig. 2 illustrates the container pressures induced by thermal runaway (TR) in five different battery compositions. The maximum pressures are in the following sequence: NCM9 0.5 0.5 > NCM811 > NCM622 > NCM523 > NCM111. The maximum pressure escalation rate is notably observed in the NCM811 battery, reaching approximately 62.8 kPa m s⁻^1^, which is approximately 30 times that of the NCM111 battery with the minimum pressure escalation rate. The quantity of gas generated during thermal runaway, calculated using the ideal gas equation (1), follows a normalized numerical order once the gas pressure stabilizes: NCM111 (0.107 mol/Ah) ≈ NCM9 0.5 0.5 (0.102 mol/Ah) ≈ NCM622 (0.100 mol/Ah) ≈ NCM811 (0.086 mol/Ah) ≈ NCM523 (0.084 mol/Ah).

**Fig. 3:**
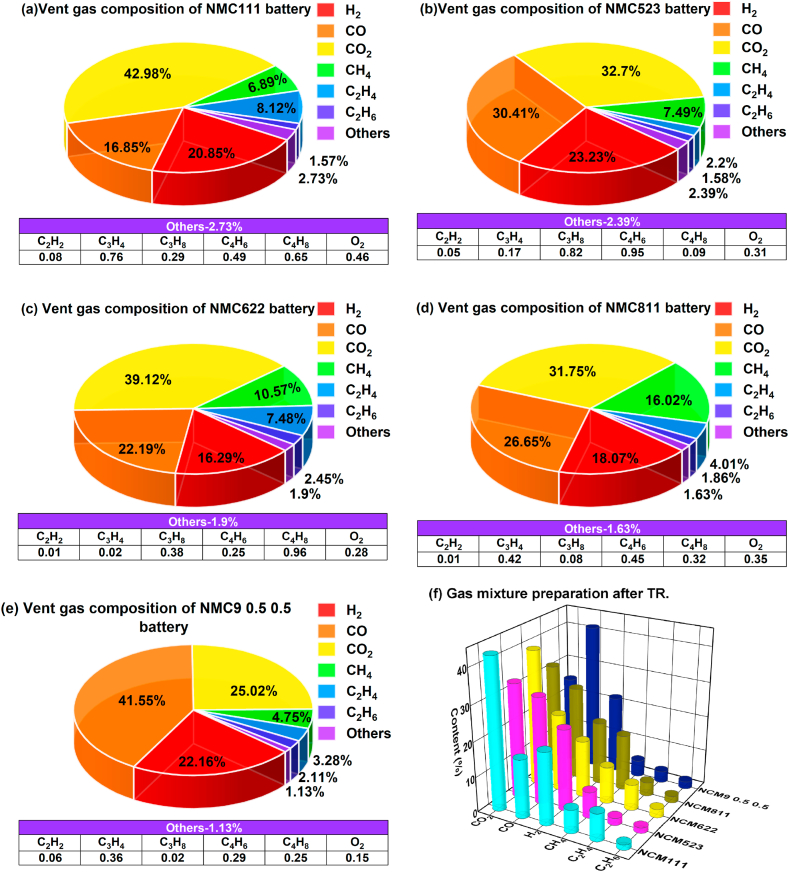
Gas composition during battery thermal runaway.

Fig. 3 summarizes the gas compositions of batteries featuring diverse cathode materials following TR experiments. Gas composition percentages are reported, excluding nitrogen. It was observed that the gas components generated by ternary NCM batteries during TR with a volume exceeding 1 % include CO_2_, CO, H_2_, CH_4_, C_2_H_4_, and C_2_H_6_, collectively constituting over 95 % of the total gas. Among the different types of batteries, the top three gases produced are CO_2_, CO, and H_2_. The percentage of CO_2_ gas composition varies from 25 % to 43 %, indicating significant differences in CO_2_ production levels. CO concentrations are in the following sequence: NCM 9 0.5 0.5 (41.55 %) > NCM 523 (30.41 %) > NCM 811 (26.65 %) > NCM 622 (22.19 %) > NCM111 (16.85 %). This order signifies notable variations in CO generation levels. The total CO + CO_2_ content in the battery is 62.49 ± 4.09 %. The findings reported in previous studies [5,23], where CO + CO_2_ levels fluctuate by approximately 60 %, are consistent with the results of this study. The volume percentage of H_2_ ranges from 16.29 % to 23.23 %. Fig. 3 presents a comparative analysis of the predominant gas components produced during TR for various battery types.

### 3.2 Combustion characteristics of the gases emitted

Publicly available literature indicates that ejecta resulting from thermal runaway comprises a three-phase mixture of gas, solid, and liquid components. Understanding the explosive limit of such mixtures presents a complex challenge. To date, most literature [32,33] has focused solely on the explosion limit of gas components. Despite these advancements, research specifically addressing the explosive limits of thermal runaway ejecta and three-phase mixtures remains elusive. In this study, only the influence of the gaseous phase was considered.

**Fig. 4:**
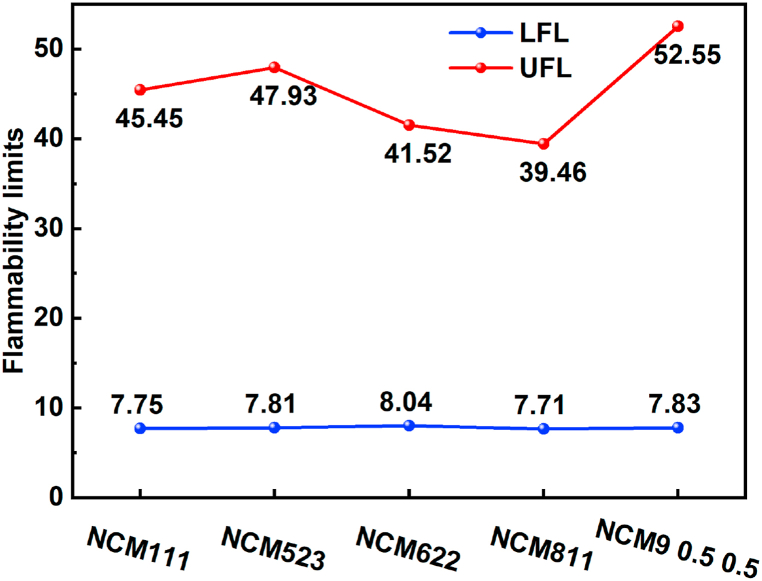
UFL and LFL values of battery TR gas.

The flammability limits and maximum explosion overpressures are important parameters for assessing the risk of gas explosions in lithium-ion batteries used for energy storage [5]. These limits, known as the upper flammability limit (UFL) and lower flammability limit (LFL), and the explosion overpressures of emitted gases are determined by mixing the gases with air. Ignition can occur during experiments when the ratio of combustible gas to air falls between the LFL and UFL, while ignition does not occur outside this range. Various methods for estimating the flammability limits of gas mixtures have been discussed in the literature [22]. In a study conducted by the Federal Aviation Administration in the United States [22], Le Chatelier's law (Equation (2)) was used to calculate the flammability limits of gas mixtures based on the volume and mole fraction values of individual fuel species. Table 2 provides the flammability limits of individual fuels. Fig. 3 shows that the gases emitted by each type of battery during thermal runaway include noncombustible carbon dioxide. In this study, when addressing gas mixtures containing inert gases, the methodology outlined in the literature [22] was followed to combine carbon dioxide and hydrogen. The flammability limits for the ratio of carbon dioxide to hydrogen could be found in the Jones chart [22], and Equation (2) was subsequently applied to calculate the flammability limits of the gas mixture. The results obtained are presented in Fig. 4. Although there is a minor error of approximately 7 % associated with this method when calculating the upper flammability limit of multicomponent gas mixtures [23], This formula has gained wide acceptance and significant reference value in engineering applications.

(2) $$L=\frac{1}{\sum_{i=1}^{n}\frac{x_{i}}{L_{i}}}\times 100\%$$

where *L* represents the explosion limit of the combustible gas mixture in percentage (%); $L_{i}$ denotes the explosion limit of an individual component in the mixed gas in percentage (%); and $x_{i}$ signifies the volume fraction of each component in the mixed gas in percentage (%).

**Table 2:** Limits of combustibility for common flammable gases [23].

Gas	UFL	LFL
CO	74	12.5
H_2_	75.6	4
CH_4_	15	5
C_2_H_4_	36	2.7
C_2_H_6_	13	2.9
C_2_H_2_	72.3	2.4
C_3_H_4_	11.7	1.7
1.3–C_4_H_6_	16.3	1.1
C_3_H_6_	10.3	2.4
C_3_H_8_	9.5	2.2
C_4_H_8_	10.0	1.8

The relationship between ignition limits and gas concentrations can be analyzed by integrating the information from Figs. 3 and 4, and Table 2. It is evident that the lower explosion limit (LFL) for various battery-emitted gases falls within a relatively consistent range of approximately 7.88 ± 0.16 %. This finding supports previous studies [5,23]. The LFL shows a positive correlation with the concentrations of CO_2_ and CO, with CO_2_ having the most significant influence. Conversely, the LFL demonstrates a negative correlation with the concentrations of H_2_, CH_4_, and C_2_H_4_, with H_2_ having the strongest impact. Conversely, the upper explosion limit (UFL) exhibits a positive correlation with the concentrations of CO_2_, CO, and H_2_, with H_2_ exerting the greatest influence. In contrast, the UFL is negatively correlated with the concentrations of CH_4_ and C_2_H_4_, with CH_4_ having the most notable effect.

Considering the significantly lower explosive lower limit (LFL) of H_2_ relative to that of CO, an increase in H_2_ concentration substantially decreases the LFL and significantly increases the UFL. This expansion of the ignition limit range greatly increases flammability hazards. Similarly, CO has a facilitating effect on expanding the ignition limit range. Conversely, CO_2_, CH_4_, and C_2_H_4_ contribute to reducing the ignition limit range, thereby decreasing flammability hazards. Among these gases, inert gas (CO_2_) has a significant influence on reducing the flammability range. Reference [34] elaborates extensively on the computation methods for parameters $c_{\text{CEG},\text{ignition}}$ and $c_{O2,\text{ignition}}$. Utilizing the outcomes depicted in Fig. 4, calculations for $c_{\text{CEG},\text{ignition}}$ (7.7–8.0) and $c_{O2,\text{ignition}}$ (9.5–12.1) were performed, accounting for variations in experimental conditions. The resulting conclusions remain consistent with those presented in Ref. [34]. The conclusions drawn from Fig. 4 are deemed to hold practical relevance for real-world applications.

While the results presented in Fig. 4 may appear irregular, it is essential to analyze them in the context of the broader experimental framework and consider the underlying complexities of the thermal runaway process. Due to functional constraints inherent in the gas composition measuring instrument, it is unable to detect all gas components. Public literature [34] suggests that thermal runaway can generate over 30 types of combustible gases. However, the instrument's inability to detect certain components means that the impact of these undetected gases on the explosion limit remains unexplored.

The explosion index, determined by the value of K, measures the destructiveness of an explosion. K represents the highest explosion index observed through experimental testing with varying concentrations of reactants. This index is directly related to the maximum rate at which the pressure increases [5]. The rate at which the indoor pressure increases due to battery venting is closely related to the size of the enclosure, and the explosion index of the gas mixture can be calculated using Equation (3).

(3) $$K={\left(dp/dt\right)}_{\max}\times V^{1/3}$$

where V is the volume of the experimental chamber.

In order to facilitate the analysis and comparison, the TR results are summarized in Table 3.

**Table 3:** Thermal runaway results.

Sample Cathode	T_S,max_ (°C)	Mass Loss Rate (k)	K (kPa ⋅ m ⋅ s^−1^)	Gas Production (mol ⋅ Ah^−1^)
NCM111	540.1	35.64 %	2.22	0.107
NCM523	542.8	38.66 %	4.77	0.084
NCM622	638.1	39.18 %	6.10	0.100
NCM811	617.9	64.87 %	62.8	0.086
NCM9 0.5 0.5	650.0	65.32 %	48.3	0.102

An analysis was conducted to assess the impacts of battery capacity and experimental chamber volume on the explosion index, denoted as K.

**Fig. 5:**
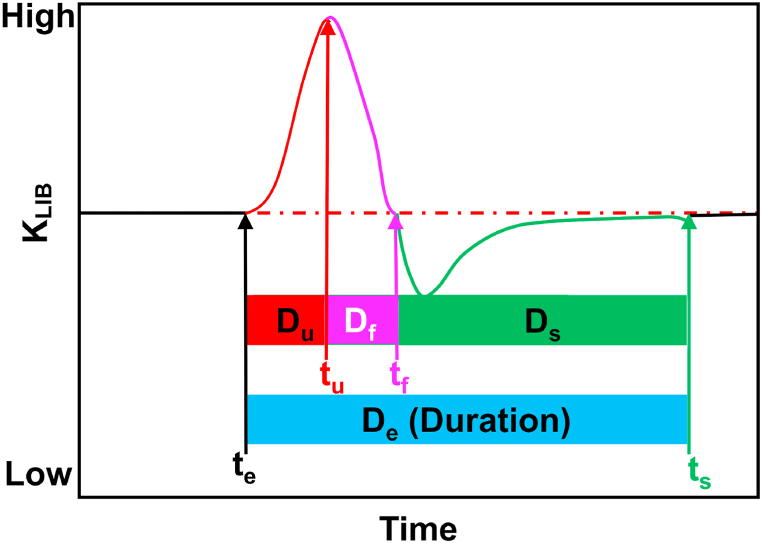
Schematic of the K_LIB_ curve [7].

First, the influence of battery capacity on the explosion index K was investigated. As depicted in Fig. 5, the results indicate that for NCM811 and NCM9 0.5 0.5, the range of K values falls within 55 ± 7.5 kPa ⋅ m ⋅ s^−1^. For NCM111, NCM523, and NCM622, the range of K values is 4.15 ± 1.95 kPa ⋅ m ⋅ s^−1^. With an approximately fivefold increase in battery capacity, the explosion index K increases by approximately elevenfold.

Furthermore, when considering the impact of the experimental chamber volume on the explosion index K, a comparison was made with a related experimental study [5], in which the scholars conducted experiments under similar fixed-volume inert atmospheric conditions. In the mentioned study, the experimental chamber had a volume of 12 L, and the maximum explosion index K values were as follows: NCM 111 = 2.0 MPa ⋅ m ⋅ s^−1^, NCM 523 = 2.04 MPa ⋅ m ⋅ s^−1^, NCM 622 = 1.67 MPa ⋅ m ⋅ s^−1^, and NCM 811 = 1.75 MPa ⋅ m ⋅ s^−1^. In this study, with a reduction in experimental chamber volume by a factor of 83, the explosion index K exhibits significant increases of 909-fold (NCM 111), 408-fold (NCM 523), 269-fold (NCM 622), and 36-fold (NCM 811) relative to the study with the larger chamber volume.

### 3.3 Idealized treatment of pressure increase rates

The rate of pressure change and the pressure values within the chamber can serve as indicators of gas generation intensity during TR when experiments are conducted in a confined space. The pressure increase rate curve during battery TR exhibits several characteristic features. Initially, a swift surge in the pressure increase rate occurs within the sealed chamber. This phenomenon is mainly attributed to the opening of the battery safety valve, enabling the release of gas from inside the battery into the sealed chamber, resulting in a rapid increase in chamber pressure, with the pressure increase rate reaching its peak. Subsequently, the pressure increase rate shifts from positive to negative, signifying the attainment of the maximum internal pressure within the sealed chamber. The decline in pressure may be attributed to the liquefaction of thermal gases within the sealed chamber, leading to a reduction in pressure [7].

Given that the experiments were conducted within a confined environment, the pressure change rate and pressure values within the chamber serve as quantitative indicators of the gas generation intensity during TR. To analyze the ejection process in lithium-ion batteries, we followed the calculation method outlined in Ref. [7]. Computational Equation (4) was employed to define the characteristic time intervals.

1) The time when K_LIB_ exhibits a rapid increase is defined as the onset of ejection ($t_{e}$).
2) The time when the K_LIB_ reaches its maximum value is defined as the conclusion of high-speed ejection ($t_{u}$).
3) The period during which K_LIB_ transitions from positive to negative values is defined as the termination of rapid ejection ($t_{f}$).
4) The time when the K_LIB_ returns to its initial pre-ejection fluctuation state is defined as the conclusion of slow-speed ejection ($t_{s}$).

The durations corresponding to the key times defined above are as follows.

1) $D_{u}$ is the duration of high-speed ejection, referring to the time interval from $t_{e}$ to $t_{u}$.
2) $D_{f}$ is the duration of rapid ejection, referring to the time interval from $t_{u}$ to $t_{f}$.
3) $D_{s}$ is the duration of slow ejection, referring to the time interval from $t_{f}$ to $t_{s}$.
4) $D_{e}$ is the overall ejection duration, described as the time interval from $t_{e}$ to $t_{s}$.

According to the aforementioned definitions, the LIB ejection process is categorized into stages of ultrahigh-speed ejection, rapid ejection, and slow ejection, as illustrated in Fig. 5.

(4) $$K_{\text{LIB}}=\left(dp/dt\right)V^{1/3}$$

where V is the volume of the experimental chamber.

The pressure vent of a lithium-ion battery ruptures before TR. In this study, the values of the K_LIB_ calculated for the gas eruption when the safety valve ruptures before thermal runaway are very small. The release of numerous substances is primarily concentrated during thermal runaway (TR). Therefore, the main investigation of TR eruption time division is illustrated in Fig. 5 for the time range of -20–60 s.

**Fig. 6:**
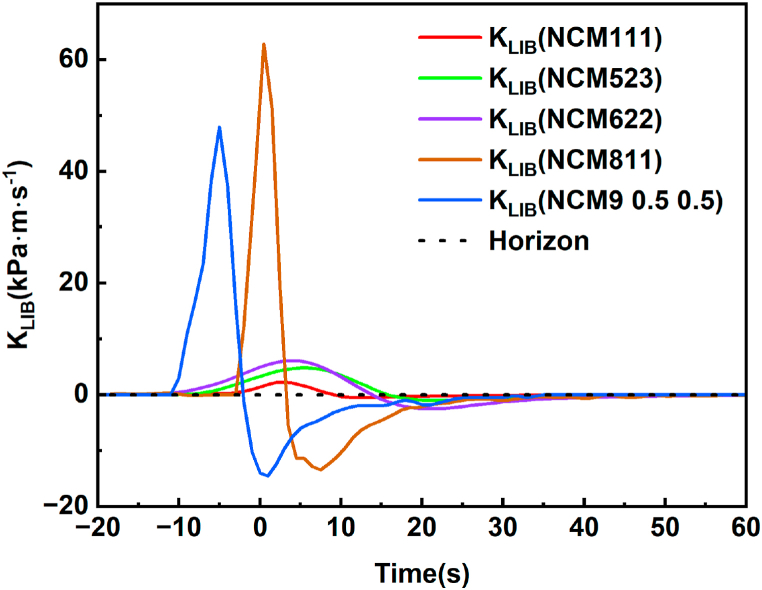
Variations in P and K_LIB_ vs. time (drawing of partial enlargement: 20–60 s).

The battery experiences a prolonged duration from heating to the ejection of TR products. In contrast, the temporal evolution of the ejection process is considerably reduced. Consequently, the K_LIB_ curve pertaining to the ejection process is the primary focus in Fig. 5. In Fig. 5, data points with three consecutive values greater than zero in the K_LIB_ are considered valid. The outcomes corresponding to various time points are summarized in conjunction with Fig. 5, Fig. 6, and these results are consolidated in Table 4.

**Table 4:** Timing of the eruption phases.

Sample Cathode	$D_{u}\left(s\right)$	$D_{f}\left(s\right)$	$D_{s}\;\left(s\right)$	$D_{e}\;\left(s\right)$
NCM111	13.5	6.2	30.5	51.2
NCM523	14.2	9.4	36.3	59.9
NCM622	15.8	10.1	43.2	69.1
NCM811	3.6	2.5	26.5	32.6
NCM9 0.5 0.5	6.0	3.1	25.3	34.3

Based on the ejection time results presented in Tables 4 and it is possible to estimate the average mass loss per second (A) during battery thermal runaway using Equation (5). A_NCM111_ = 5.92 g ⋅ s^−1^, A_NCM523_ = 5.72 g ⋅ s^−1^, A_NCM622_ = 4.89 g ⋅ s^−1^, A_NCM811_ = 68.05 g ⋅ s^−1^, and A_NCM9 0.5 0.5_ = 46.66 g ⋅ s^−1^.

(5) $$A=\frac{m\cdot k}{D_{e}}$$

where A is the average mass loss per second during battery thermal runaway, expressed in grams per second, g ⋅ s^−1^; m is the mass of the battery before thermal runaway, g; and k is the mass loss rate.

## 4 Conclusions and summary

The aim of this study was to examine the gas generation profiles of diverse NCM batteries during TR. Experiments took place in a fixed-volume inert atmosphere containing 99 % N_2_. Information regarding TR temperatures, gas evolution characteristics, and combustion behaviors of distinct batteries were gathered. The principal findings can be summarized as follows.

(1) Comparison was made with experimental papers authored by others [5,23,31,35,36] (a total of 5 papers involving 20 battery specimens). The batteries used in these papers and in the present study had the same cathode materials and shapes, and the experimental conditions were consistent. TR experiments were performed in an inert and fixed-volume environment. The normalized gas generation rate for TR in the batteries reported in these 5 papers was found to be 0.1 ± 0.03 mol·Ah⁻^1^, which closely aligned with the results of this study (0.10 ± 0.02 mol·Ah⁻^1^). This finding suggested that the maximum capacities of prismatic ternary batteries negligibly impacted the normalized gas generation rate following thermal runaway.
(2) Based on a comprehensive assessment of battery temperature, ejection duration, and chamber pressure increases during the experimental procedure, the hierarchy of thermal runaway hazards was established as follows: NCM811>NCM9 0.5 0.5>NCM622>NCM523>NCM111. In this study, NCM811 emerged as the most hazardous cathode material used in lithium-ion batteries, surpassing the hazard level of NCM9 0.5 0.5 batteries. It was generally understood that the thermal runaway hazard escalated as the proportion of nickel in the battery anode increased. In the present investigation, this outcome was attributed to the relatively high capacity of the battery specimens used, and the experiments being conducted within a 1000-L fixed-volume inert environment. However, it should be noted that alterations in chamber volume, and according to a comparison conducted in ambient air, could result in NCM9 0.5 0.5 exhibiting a higher level of hazard than NCM811.
(3) The study demonstrated that reducing the chamber volume or increasing the battery capacity resulted in a high explosion index, referred to as K. In this study, the influences of nickel, cobalt, and manganese in the cathode material on the explosion index were not considered. However, a fivefold increase in capacity led to an approximately elevenfold increase in the explosion index. Furthermore, under similar conditions of cathode material and battery capacity as those applied in Ref. [5], reducing the chamber volume by 83-fold corresponded to 408-fold and 269-fold increases in the explosion indices of NCM523 and NCM622, respectively.
(5) When comparing the results of this study to similar experimental conditions in the literature [5,23], it could be observed that the flammable lower limits (LFLs) of gases emitted during the venting of different types of NCM batteries ranged within 8 ± 1.8 %, showing minimal variation. Conversely, the flammable upper limits (UFLs) had relatively wide ranges, spanning from 30 % to 60 %.

It was established that gases emitted from various types of prismatic ternary lithium-ion batteries posed substantial combustion and explosion risks. The explosion parameters acquired for five battery chemical compositions offered valuable insights for enhancing the safety of electrochemical energy storage facilities. However, there were three limitations to this research. First, this experiment did not provide mathematical principles or statistical models regarding the influences of prismatic ternary lithium-ion battery capacity and experimental chamber volume on explosion indices. Second, this study did not address the impacts of battery capacity and constant-volume experimental chamber volume on the duration of venting during thermal runaway. Third, the experiments did not achieve the goal of altering only a single variable to study its effects on gas composition, jetting time, explosion index, and other factors.

## Data sstatement

The datasets generated and/or analyzed in this study are not publicly available due to [REASON WHY DATA ARE NOT PUBLIC], but they are available from the corresponding author upon reasonable request.

## CRediT authorship contribution statement

**Ningning Wei:** Writing – review & editing, Writing – original draft, Visualization, Validation, Supervision, Software, Resources, Project administration, Methodology, Investigation, Formal analysis, Data curation, Conceptualization. **Fengqin Zhang:** Resources. **Wei Zhang:** Project administration, Methodology. **Xin Li:** Supervision, Funding acquisition.

## Declaration of competing interest

The authors declare that they have no known competing financial interests or personal relationships that could have appeared to influence the work reported in this paper.

## Nomenclature

T_S_: Cell side surface center temperature, °C
T_A_: Experimental chamber ambient temperature, °C
D_e_: Eruption duration
D_u_: Ultrafast eruption duration
D_f_: Fast eruption duration
D_s_: Slow eruption duration
TR: Thermal runaway
t_u_: End of ultrafast eruption
t_f_: End of fast eruption
t_S_: End of slow eruption
t_e_: Start of eruption
ISO: International Organization for Standardization
LIBs: Lithium-ion battery
AEC: Constant volume adiabatic experimental chamber
t: time, s
K: Gas explosion index
K_LIB_: Battery eruption index
CEG: The cell eruption gas
$c_{\text{CEG},\text{ignition}}$: Minimum CEG concentration required for ignition
$c_{O2,\text{ignition}}$: Minimum oxygen concentration required for ignition
BTMS: Battery thermal management system
SOC: State of charge
P: Pressure, kPa
C-rate: Charge and discharge current with respect to its nominal capacity
ESS: Energy storage systems
LFL: Lower flammability limit
UFL: Upper flammability limit
A: Average mass loss per second
k: Mass loss rate
